# Supplementary material for: A billion years of evolution manifest in nanosecond protein dynamics
Source: Proc Natl Acad Sci U S A. 2024 Feb 27;121(10):e2318743121. doi: 10.1073/pnas.2318743121 (PMC10927572; doi:10.1073/pnas.2318743121)
Supplement: Supplementary file 1 — Appendix 01 (PDF) [file pnas.2318743121.sapp.pdf]

# Supporting Information for

## A billion years of evolution manifest in nanosecond protein dynamics

**Philipp J. Heckmeier, Jeannette Ruf, Charlotte Rochereau, and Peter Hamm**

**Corresponding Author: Philipp J. Heckmeier.**
**E-mail: philipp.heckmeier@chem.uzh.ch**

### This PDF file includes:

Supporting text
Figs. S1 to S7
Tables S1 to S2
SI References

## Supporting Information Text

## Lifetime Analysis

In transient infrared experiments, we obtained homolog-specific kinetic footprints in the form of two-dimensional data sets as a function of probe frequency $\omega_i$ and pump-probe delay time $t_j$, described in the Methods section in the main article. Complementing a global multiexponential fitting approach, we applied a lifetime analysis to the 2D dataset (1) in order to examine the correctness of the fitting.

In the lifetime analysis, the time constants $\tau_k$ are not considered as fitting parameters thus remain unchanged, distributed equidistantly on a logarithmic scale with 10 terms per decade. Exclusively the amplitudes $a(\omega_i, \tau_k)$ are treated as fitting parameters. Regularization of the fit is achieved by applying a maximum entropy method (1–3). This results in lifetime spectra exhibiting distinct features of increased activity (Fig. S5A), that are colored red or blue corresponding to positive or negative amplitudes, respectively. To gauge dominant time scales of increased dynamic activity, we determined an "averaged dynamical content" (4) by averaging over the probe frequencies of the spectrum:

$$D(\tau_k) = \sqrt{\sum_i a(\omega_i, \tau_k)^2}. \quad [1]$$

Analyzing ten kinetic footprints with lifetime analysis (Fig. S5) yields three dominant phases of protein response, similar to the global multiexponential fitting. The early stage is characterized by a shallow maximum, the mid phase by a dominant and – in most cases – well defined peak, the late phase by diffuse and dispersive maxima.

The observed diffuse kinetics might be an indicator for a stretched exponential behavior, however, lifetime analysis also has a tendency for overfitting. If the kinetics is stretched exponential, then global fitting would reveal an average.

## PDB files

This section contains the list of all PDB files computed using AlphaFold and RosettaFold. The corresponding files can be accessed in the zipped folders provided alongside this publication.

### AlphaFold.

- *H.sapiens*: Homo_21982_unrelaxed_rank_001_alphafold2_ptm_model_1_seed_000.pdb
- *M.musculus*: Mus_289c8_2_unrelaxed_rank_001_alphafold2_ptm_model_1_seed_000.pdb
- *B.taurus*: Bos_1af98_0_unrelaxed_rank_001_alphafold2_ptm_model_5_seed_000.pdb
- *G.gallus*: Gallus_2ef02_unrelaxed_rank_001_alphafold2_ptm_model_5_seed_000.pdb
- *A.mississippiensis*: Alligator_1af98_unrelaxed_rank_001_alphafold2_ptm_model_5_seed_000.pdb
- *X.laevis*: Xenopus_f5770_unrelaxed_rank_001_alphafold2_ptm_model_4_seed_000.pdb
- *D.rerio*: Danio_71ee0_unrelaxed_rank_001_alphafold2_ptm_model_3_seed_000.pdb
- *P.marinus*: Petromyzon_bb260_unrelaxed_rank_001_alphafold2_ptm_model_3_seed_000.pdb
- *L.unguis*: Lingula_e537d_unrelaxed_rank_001_alphafold2_ptm_model_1_seed_000.pdb
- *H.vulgaris*: Hydra_0c367_unrelaxed_rank_001_alphafold2_ptm_model_3_seed_000.pdb
- *O.anatinus*: Ornithorhynchus_20dc8_unrelaxed_rank_001_alphafold2_ptm_model_5_seed_000.pdb
- *O.cincta*: Orchesella_530a8_unrelaxed_rank_001_alphafold2_ptm_model_4_seed_000.pdb
- *A.planci*: Acanthaster_657f6_0_unrelaxed_rank_001_alphafold2_ptm_model_5_seed_000.pdb

### RosettaFold.

- *H.sapiens*: homo_robetta.pdb
- *M.musculus*: mus_robetta.pdb
- *B.taurus*: bos_robetta.pdb
- *G.gallus*: gallus_robetta.pdb
- *A.mississippiensis*: alligator_robetta.pdb

- *X.laevis*: xenopus_robetta.pdb
- *D.rerio*: danio_robetta.pdb
- *P.marinus*: petromyzon_robetta.pdb
- *L.unguis*: lingula_robetta.pdb
- *H.vulgaris*: hydra_robetta.pdb
- *O.anatinus*: ornithorhynchus_robetta.pdb
- *O.cincta*: orchesella_robetta.pdb
- *A.planci*: acanthaster_robetta.pdb

Additionally, RMSD values and TM scores for all combinations of predicted structures can be found in: rosettafold-data.csv (RosettaFold) and alphafold-unrelaxed-data.csv (AlphaFold).

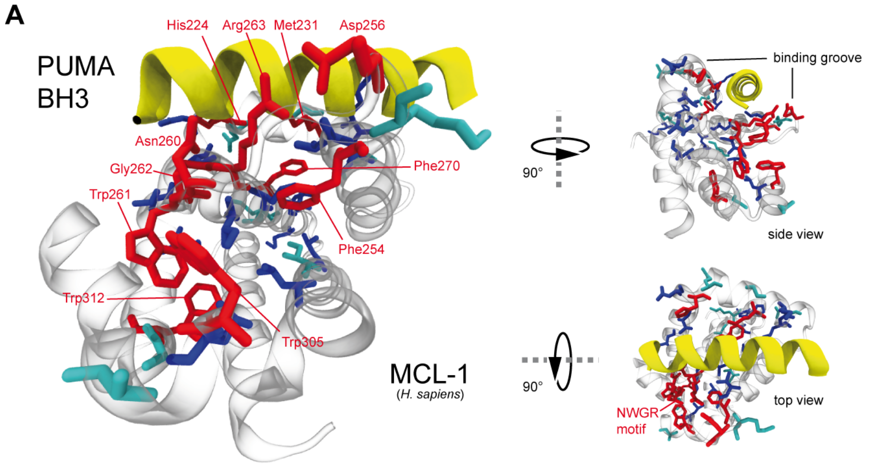


```
H.sapiens            -EDELYRQSLEIISRYLREQATGAKDTKPM---------G-------RSGATSRKALETL     43
M.musculus           -EDDLYRQSLEIISRYLREQATGSKDSKPL---------G-------EAGAAGRRALETL     43
B.taurus             -EDELYRQSLEIISQYLREQATGAKDAKPL---------G-------GSGTTSRKALETL     43
G.gallus             -PDELRQESLELILRYLREAAGEA---EPGVKKLFPGLLGGPGRPGRASSAVMEKALETL     56
A.mississippiensis   -EDELRRASLALISRYLREAAGAV-DSGPGGKNLFKGLLGAGGR---AAEPGLERALDTL     55
X.laevis             -KDGLYMDTQQLILAFYRVYSGEESGELE--------------ASCLLQHGVHHKALETL     45
D.rerio              -YAELERDTRQLLLDFYRTHTGMC-----------------------PVDRKLHHAIPTM     36
P.marinus            NDDALTVETANFITGYMAHLTPTE---------------------QPPHQQQLSSASELL     39
L.unguis             -EEHLPS-------SAI------AKTAKEIAHDVLFWTV------GYNNSKPNDTYSKTM     40
H.vulgaris           ------------SDTPINFNTPEAKIAKEVAYQLIQKKTG-----KNIKNQQIRKETELM     43
                                                                                :

H.sapiens            RRVGDGVQRNHETAFQGMLRKLDIKNEDD-VK-SLSRVMIHVFSDGVTNWGRIVTLISFG     101
M.musculus           RRVGDGVQRNHETAFQGMLRKLDIKNEGD-VK-SFSRVMVHVFKDGVTNWGRIVTLISFG     101
B.taurus             RRVGDGVQRNHETAFQGMLRKLDIKNEDD-VK-SLSRVMVHVFSDGVTNWGRIVTLISFG     101
G.gallus             RRVGDGVMQKHELAFQGMLRKLEIKKEDD-LQ-AVCEVAAHVFNDGVTNWGRVVTLISFG     114
A.mississippiensis   RRVGDGVLQKHQLAFQGMLRKLEIRNEED-LQ-SVSAVATHMFSDGITNWGRVVTLISFG     113
X.laevis             LRVGGEIIEKHHMAFTGMLQRLSIHSRED-LQ-KLSEVPALVFNDGVTNWGRIVTVISFG     103
D.rerio              KRVVDNILVKHQIAYKGMIQRLQLDSQPASLD-FIRCIASTMFKDGVTNWGRIASLVAFG     95
P.marinus            KSMGDSLLEQHRAAISGMVSRVEAQTDPEELRGSALGVAREMFSDGHMNWGRVVALVVFC     99
L.unguis             RRLVTEILEKHKITFNGMMNKLDLSELNG-K-ETVNGCLEHIFTDHEVNWGRVVTIYAFG     98
H.vulgaris           MRLVEEIEIKHELFLSNMCKRLNLNTENA--QSMFHTIAEEIFSDGI-NWGRIIVLYAFA     100
                       :   :  :*.    .*  ::.                  :*.*   ****:  :  *

H.sapiens            AFVAKHLKTINQES--SIEPLAESITDVLVRTKRDWLV-KQRGWDGFVEFFHVEDLEGG      157
M.musculus           AFVAKHLKSVNQES--FIEPLAETITDVLVRTKRDWLV-KQRGWDGFVEFFHVQDLEGG      157
B.taurus             AFVAKHLKSINQES--CIEPLAESITDVLVRSKRDWIV-KQRGWDGFVEFFHVEDLEGG      157
G.gallus             AFVAKHLKSINQEK--CITSLAGIITDALVSSKREWLM-SQGGWEGFVDFFRVEDLESS      170
A.mississippiensis   AFVVKHLKSINQEN--CINTLAGIITDLLVTDKRDWLV-SQRGWEGFVEFFHVEDLEGS      169
X.laevis             AFVAKHLKSLNLED--CIGVLAEHFTQFLMMSKKDWII-QEKGWDGFVDFFHIEDYESG      159
D.rerio              AVVCSRLKELQQDQ--CVERVAEQISSYLTSEQQDWIL-KNKSWHGFVEFFHQEDVESV      151
P.marinus            ALLVKRLKASGRLSDAEARAVADEVAAFLVSSRKAWFV-QQGGW--------------      142
L.unguis             SRLMKHCQENDMNT--LMDKLPDYVGEYVATKLGPWIH-KQGGWDTFVASFPDKNQMEN      154
H.vulgaris           GKIAEHCRKSNDEQ--IIDKIALWVAGFVA-KKSSWIRDSGRGWNGFIENFDVKQDSWV      156
                     . : .: :            :   .   :      *:  .  .*
```

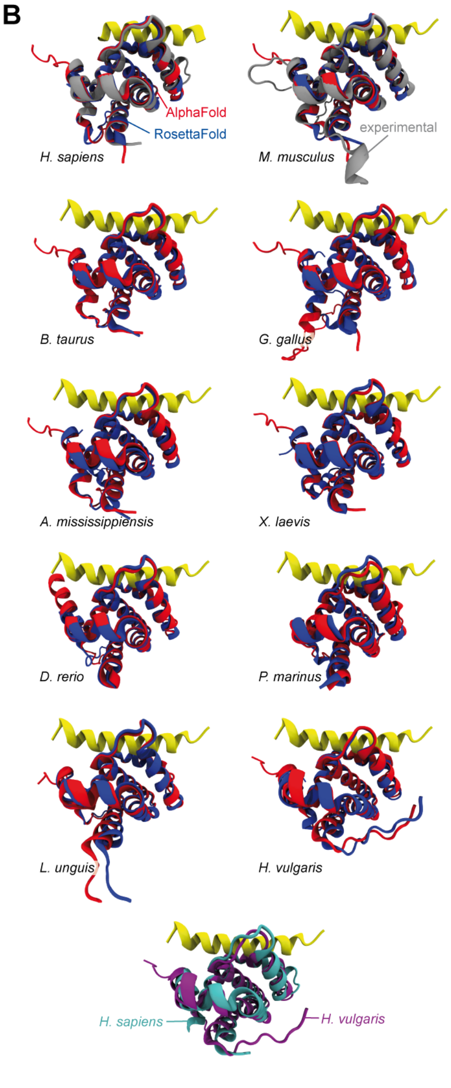


**Fig. S1.** (A) Location of the conserved residues in MCL-1 (PDB: 6QFM) according to a multiple sequence alignment of MCL-1 homolog selection. The residues in the structure are given with their position in *Homo sapiens*. The alignment was generated using Clustal Omega (EMBL-EBI)(5). "*", red: positions with a completely conserved residue. ":", blue: positions with high conservation between groups of strongly similar properties. ".", cyan: conservation between groups of weakly similar properties. The highlighted NWGR motif is characteristic for the BH1 domain of MCL-1(6) and present in all investigated homologs. (B) Structural alignment of predicted AlphaFold (red) and RosettaFold (blue) structures for the investigated MCL-1 homologs. Experimental structures (grey) were only available for *Homo sapiens* and *Mus musculus*. The complexed PUMA BH3 is displayed in yellow and was taken from the corresponding experimental structures. For homologs without experimental structures, the structure of PUMA BH3 originally from *Mus musculus* is displayed representatively. The structures at the bottom show a comparison between the experimental MCL-1 structure of *Homo sapiens* (6qfm, cyan) and the AlphaFold predicted structure of *Hydra vulgaris* (purple), two species which exhibit a evolutionary separation by more than 700 million years.

 

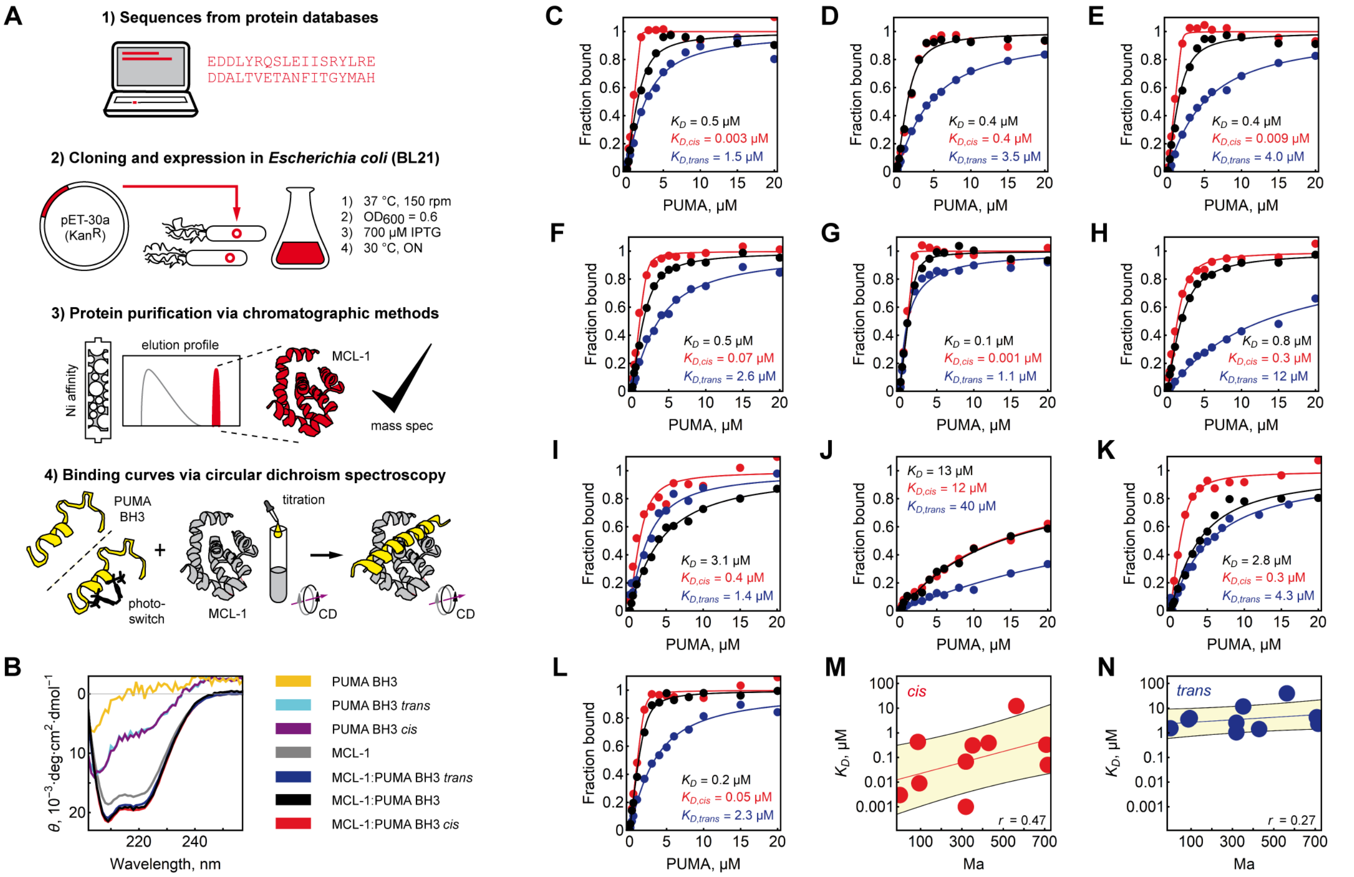


**Fig. S2.** The binding affinities of ten MCL-1 homologs. (A) Generation of ten MCL-1 homologs for spectroscopic experiments. (B) Circular dichroism spectroscopy of the MCL-1/PUMA BH3 complex. PUMA BH3 is intrinsically disordered and only assumes an $\alpha$-helical shape when bound to MCL-1. When linked to an azobenzene photoswitch, PUMA BH3 is destabilized in the *trans*-state of the photoswitch moiety. Binding curves for (C) *Homo sapiens*, (D) *Mus musculus*, (E) *Bos taurus*, (F) *Gallus gallus*, (G) *Alligator mississippiensis*, (H) *Xenopus laevis*, (I) *Danio rerio*, (J) *Petromyzon marinus*, (K) *Lingula unguis*, and (L) *Hydra vulgaris*. The binding affinity in *cis*-state (M) and *trans*-state (N) against the evolutionary divergence in million years, Ma. Yellow, linear fit $\pm$ standard deviation; plots are displayed with the Pearson correlation coefficient $r$.

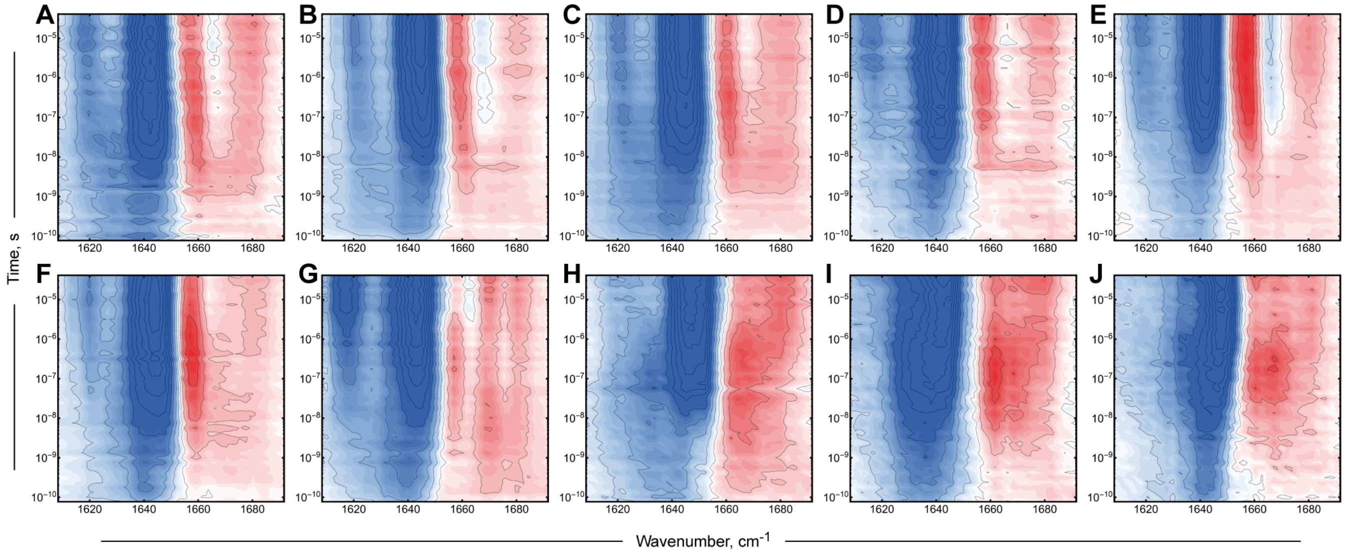


**Fig. S3.** Kinetic responses, serving as footprints, for (A) *Homo sapiens*, (B) *Mus musculus* (divergence time: 87 million years, Ma), (C) *Bos taurus* (94 Ma), (D) *Gallus gallus* (319 Ma), (E) *Alligator mississippiensis* (319 Ma), (F) *Xenopus laevis* (352 Ma), (G) *Danio rerio* (429 Ma), (H) *Petromyzon marinus* (563 Ma), (I) *Lingula unguis* (708 Ma), and (J) *Hydra vulgaris* (715 Ma). The kinetic footprints display comparable elements. We observe fine differences between closer related homologs and diverging, gradually emphasized features for farther related homologs, indicating an evolutionary timescale in which protein dynamic processes have changed.

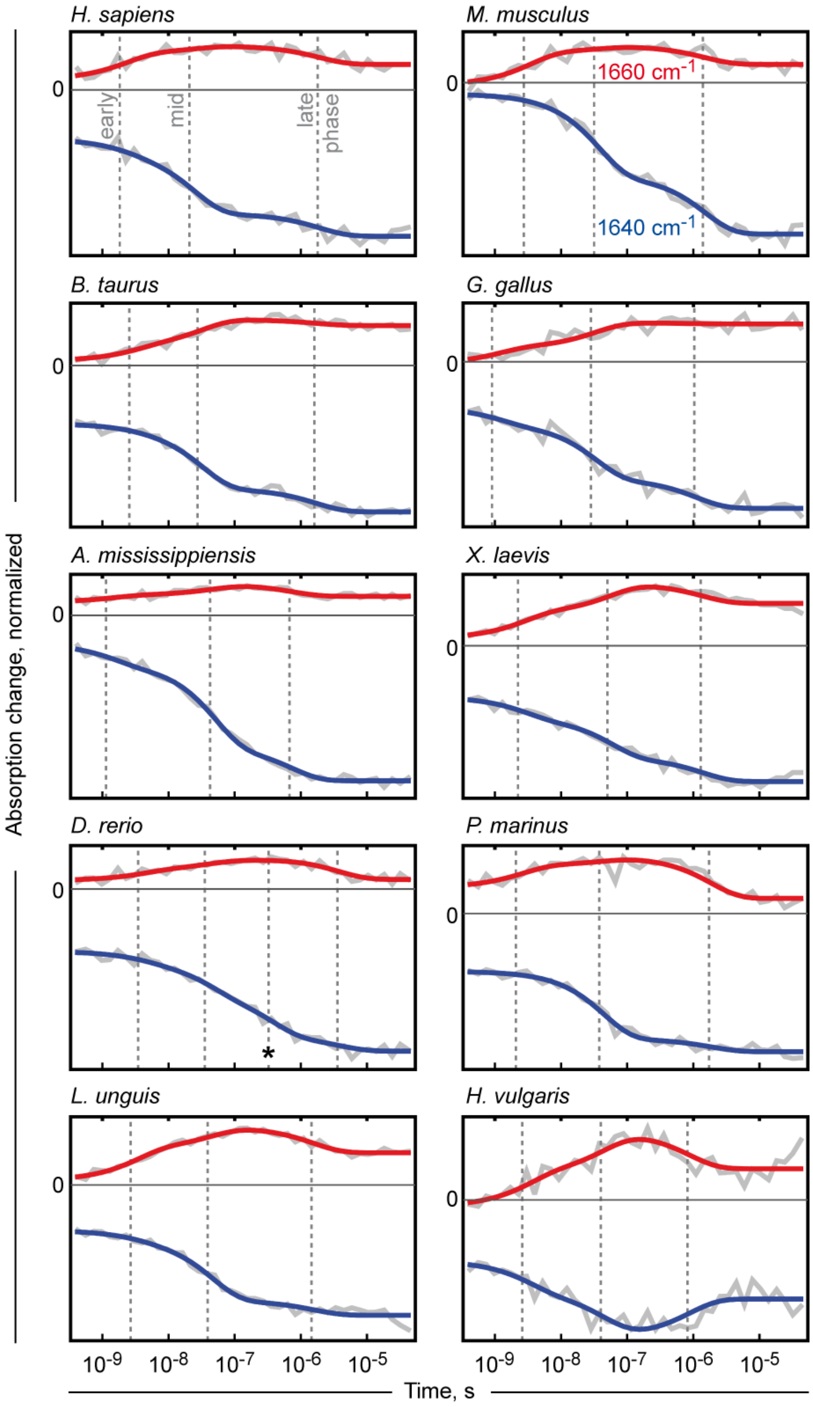


**Fig. S4.** The dynamic response upon photo-perturbation can be subdivided into an early-, mid-, and late phase, exemplified here for kinetic traces at 1640 cm$^{-1}$ and 1660 cm$^{-1}$. When analyzed with global multiexponential fitting and three time constants $\tau_{early}$, $\tau_{mid}$, and $\tau_{late}$ (given as dashed lines), the resulting fits (red/blue) are congruent with the time traces (grey). The one exception is *D. rerio*, which requires an additional fourth time constant $\tau_{D.rerio}$ = 300 ns (*) to adequately fit the data.

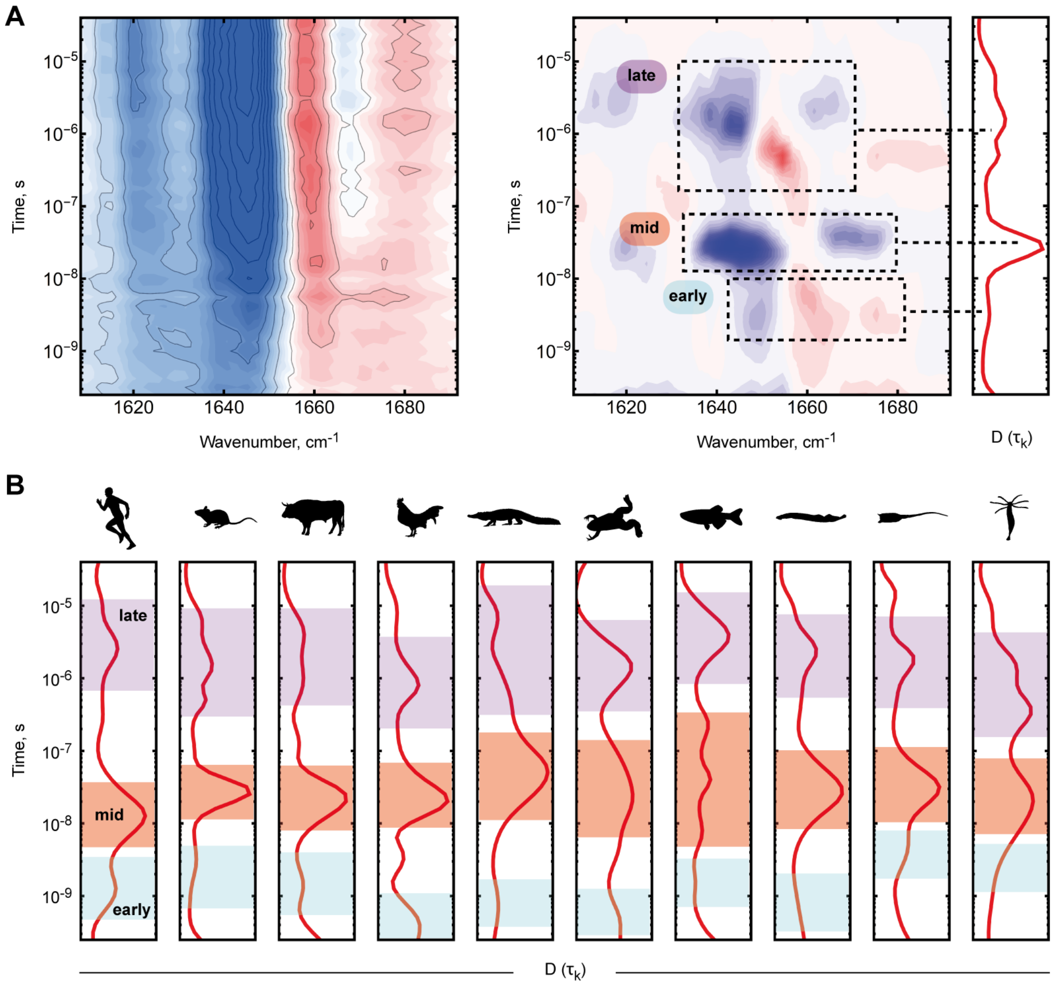


**Fig. S5.** Lifetime analysis of spectroscopic data. (A) From raw transient infrared spectra (Fig. S3) we could determine lifetime spectra and the resulting average dynamical content D($\tau_k$), here exemplarily shown for *M. musculus*. For methodical details see the section "Lifetime Analysis" in Supporting Information. An increased dynamic activity can be detected at approximately 1-3 ns (early), at 20 ns (mid), and diffusely at time scales > 200 ns (late). Maxima at 100 ps can be related to the pump–pulse duration, not reflecting a kinetic process. (B) Lifetime analysis proves that the protein dynamic response of protein complexes is dissected in an early, mid, and late phase. From left to right, dynamical contents are displayed for *H. sapiens*, *M. musculus*, *B. taurus*, *G. gallus*, *A. mississippiensis*, *X. laevis*, *D. rerio*, *P. marinus*, *L. unguis*, are *H. vulgaris* homologs.

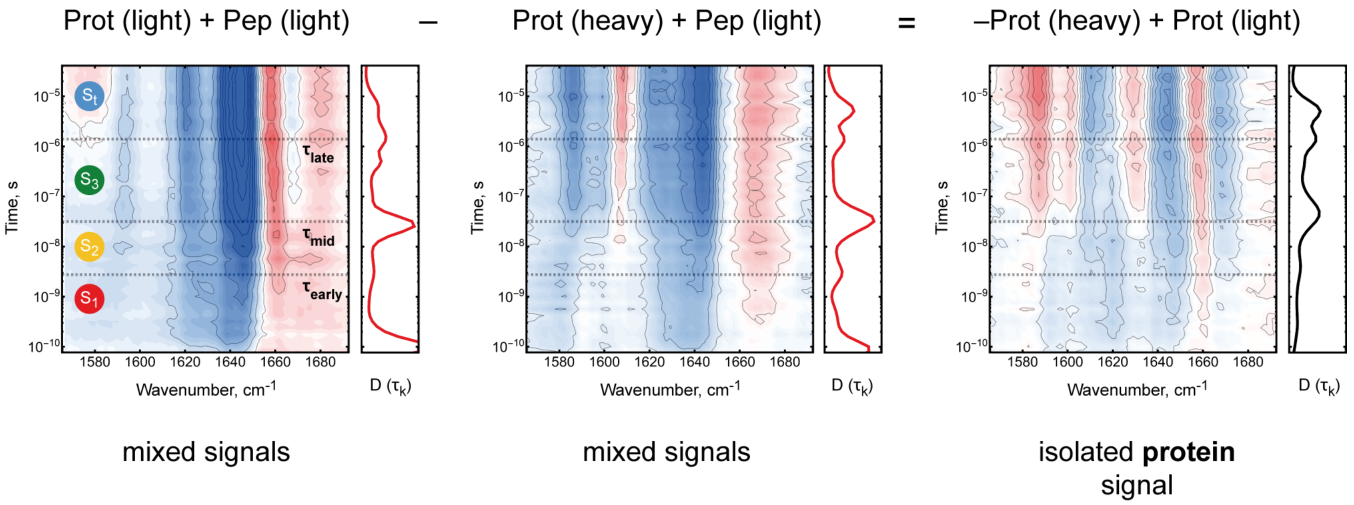


**Fig. S6.** Assignment of spectral features. Subtracting the spectrum for $^{13}$C-$^{15}$N-labelled MCL-1 protein and unlabelled BH3 peptide from the spectrum, where both protein and peptide are unlabelled, results in a double-difference spectrum (right panel). The double-difference spectrum contains signals which arise only from dynamic changes inside MCL-1 (signals from $^{13}$C-$^{15}$N-labelled protein are inverted). From the double-difference spectrum we conclude that $\tau_{early}$, which $S_2$ is populated with, can be exclusively assigned to the response of the BH3 peptide since the averaged dynamical content D($\tau_k$) for the isolated protein signal is solely increased for later time frames, $\tau_{mid}$, and $\tau_{late}$. For details on calculating D($\tau_k$), see the section "Lifetime Analysis" in the Supporting Information Text.

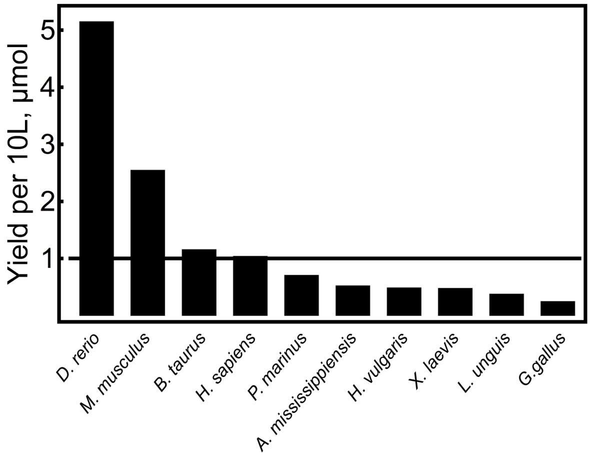


**Fig. S7.** Expression yield of various MCL-1 homologs from 10 L *Escherichia coli* culture. A yield of 1 µmol was seen crucial for spectroscopic analysis.

**Table S1. Selection of species whose MCL-1 homologs were investigated**

| | MCL-1 Uniprot ref. ID | divergence time, Ma | confidence interval, Ma |
|---|---|---|---|
| *H. sapiens* | Q07820 | 0 | - |
| *M. musculus* | P97287 | 87 | 81-91 |
| *B. taurus* | A5PJR2 | 94 | 92-97 |
| *G. gallus* | (A0A1L1RNM6)* XP_046788511.1 | 319 | 316-322 |
| *A. mississippiensis* | A0A151NDN7 | 319 | 316-322 |
| *X. laevis* | B6V6J0 | 352 | 348-356 |
| *D. rerio* | (Q1L8X3)** UPI000056A44D | 429 | 423-440 |
| *P. marinus* | - | 563 | 494-652 |
| *L. unguis* | A0A1S3IZP1_LINUN | 708 | 627-830 |
| *H. vulgaris* | A1E3K7 | 715 | 604-1250 |

*Uniprot entry (Dec 2022) has become obsolete. NCBI reference ID is given instead. **Uniprot entry (Dec 2022) has become obsolete. Uniparc ID is given instead.
The divergence times are related to *H. sapiens*.

**Table S2. Time constants representing the early-, mid-, and late phase of the dynamic response.**

| | $\tau_{early}$ ns | $\tau_{mid}$ ns | $\tau_{late}$ $\mu$s |
|---|---|---|---|
| *H. sapiens* | 1.8 | 21 | 1.8 |
| *M. musculus* | 2.7 | 31 | 1.4 |
| *B. taurus* | 2.5 | 27 | 1.6 |
| *G. gallus* | 0.9 | 28 | 1.0 |
| *A. mississippiensis* | 1.1 | 43 | 0.7 |
| *X. laevis* | 2.2 | 50 | 1.3 |
| *D. rerio* | 3.5 | 35 | 3.6 |
| *P. marinus* | 2.1 | 38 | 1.7 |
| *L. unguis* | 2.7 | 39 | 1.5 |
| *H. vulgaris* | 2.6 | 40 | 0.8 |
| *M. musculus* ($^{13}C^{15}N$) | 3.9 | 36 | 2.3 |

## References


1. D Buhrke, KT Oppelt, PJ Heckmeier, R Fernández-Terán, P Hamm, Nanosecond protein dynamics in a red/green cyanobacteriochrome revealed by transient IR spectroscopy. *J. Chem. Phys.* **153**, 245101–1–245101–12 (2020).
2. VA Lórenz-Fonfría, H Kandori, Transformation of Time-Resolved Spectra to Lifetime-Resolved Spectra by Maximum Entropy Inversion of the Laplace Transform. *Appl. Spectrosc.* **60**, 407–417 (2006).
3. VA Lórenz-Fonfría, H Kandori, Practical aspects of the maximum entropy inversion of the laplace transform for the quantitative analysis of multi-exponential data. *Appl. Spectrosc.* **61**, 74–84 (2007).
4. G Stock, P Hamm, A non-equilibrium approach to allosteric communication. *Philos. Transactions Royal Soc. B* **373**, 20170187 (2018).
5. F Sievers, et al., Fast, scalable generation of high-quality protein multiple sequence alignments using Clustal Omega. *Mol. Syst. Biol.* **7** (2011).
6. A McGriff, WJ Placzek, Phylogenetic analysis of the MCL1 BH3 binding groove and rBH3 sequence motifs in the p53 and INK4 protein families. *PLoS ONE* **18**, 1–19 (2023).
